# Flight of the bumble bee: Buzzes predict pollination services

**DOI:** 10.1371/journal.pone.0179273

**Published:** 2017-06-07

**Authors:** Nicole E. Miller-Struttmann, David Heise, Johannes Schul, Jennifer C. Geib, Candace Galen

**Affiliations:** 1 Biological Sciences Department, Webster University, St. Louis, Missouri, United States of America; 2 Department of Computer Science, Technology & Mathematics, Lincoln University, Jefferson City, Missouri, United States of America; 3 Division of Biological Sciences, University of Missouri, Columbia, Missouri, United States of America; 4 Department of Biology, Appalachian State University, Boone, North Carolina, United States of America; Indian Institute of Science, INDIA

## Abstract

Multiple interacting factors drive recent declines in wild and managed bees, threatening their pollination services. Widespread and intensive monitoring could lead to more effective management of wild and managed bees. However, tracking their dynamic populations is costly. We tested the effectiveness of an inexpensive, noninvasive and passive acoustic survey technique for monitoring bumble bee behavior and pollination services. First, we assessed the relationship between the first harmonic of the flight buzz (characteristic frequency) and pollinator functional traits that influence pollination success using flight cage experiments and a literature search. We analyzed passive acoustic survey data from three locations on Pennsylvania Mountain, Colorado to estimate bumble bee activity. We developed an algorithm based on Computational Auditory Scene Analysis that identified and quantified the number of buzzes recorded in each location. We then compared visual and acoustic estimates of bumble bee activity. Using pollinator exclusion experiments, we tested the power of buzz density to predict pollination services at the landscape scale for two bumble bee pollinated alpine forbs (*Trifolium dasyphyllum* and *T*. *parryi*). We found that the characteristic frequency was correlated with traits known to affect pollination efficacy, explaining 30–52% of variation in body size and tongue length. Buzz density was highly correlated with visual estimates of bumble bee density (*r* = 0.97), indicating that acoustic signals are predictive of bumble bee activity. Buzz density predicted seed set in two alpine forbs when bumble bees were permitted access to the flowers, but not when they were excluded from visiting. Our results indicate that acoustic signatures of flight can be deciphered to monitor bee activity and pollination services to bumble bee pollinated plants. We propose that applications of this technique could assist scientists and farmers in rapidly detecting and responding to bee population declines.

## Introduction

Declines in wild and managed bees threaten pollination services to flowering plants globally, potentially exacting costs for more than 85% of flowering plants [1], 75% of agricultural crops [2] and human health [3]. The agricultural losses alone are estimated at over $200 billion per year globally [4,5], and costs from diminished pollination services in wild ecosystems likely exceed this. Causes of pollinator declines are complex and include diminishing flower resources, habitat loss, climate change, increased disease incidence and exposure to pesticides ([3] and references therein). Given the complexity and the limited spatial and temporal scale of most studies, pinpointing the driving force(s) remains a significant challenge [6–8].

**Fig 1:**
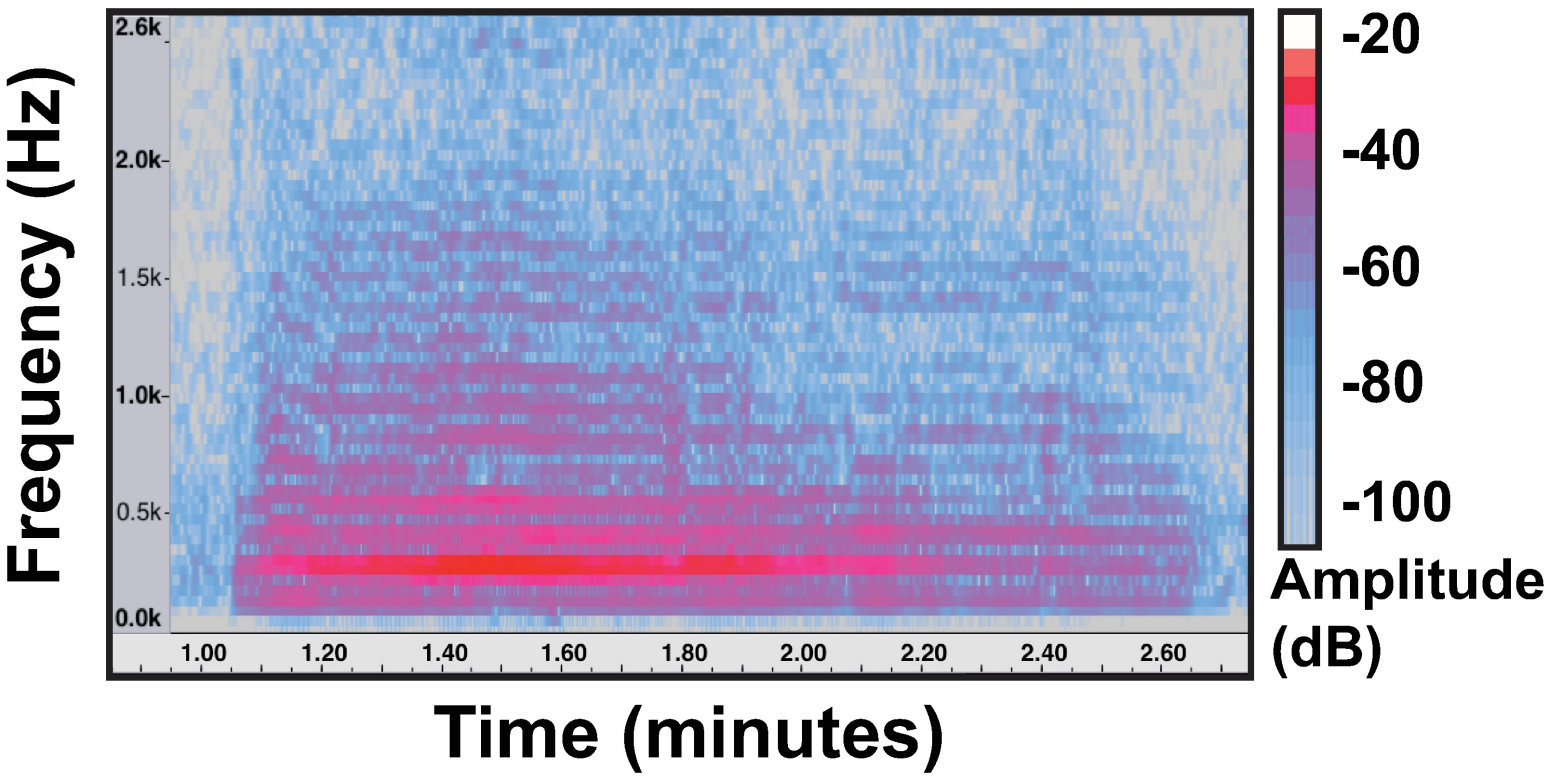
Spectrogram of a typical flight buzz by a *Bombus balteatus* queen. A spectrogram represents the energy of the audio signal within time-frequency bins, where the magnitude of each bin corresponds to the energy within a frequency range during a narrow time frame. The lowest band (approximately 175 Hz in this example) corresponds to the wing beat frequency. The sound is a harmonic series with energy at integer multiples of the wing beat frequency (known as the 1^st^ harmonic or fundamental frequency).

For over 100 years, scientists have used the sonic vibrations made by many organisms (birds, bats, frogs, insects etc.) to study communication, behavior, evolution, and population dynamics [9]. Acoustic monitoring techniques are non-invasive, less time-intensive, and can integrate expert knowledge through sound libraries. While used extensively to monitor bat and bird populations, acoustic techniques have been rarely used to monitor insects or their ecosystem services [10]. Foraging bees create vibrations (buzzes; Fig 1) between 120–400 Hz in first-harmonic frequency by rhythmic oscillation of their thorax in flight [11]. Monitoring bees in flight captures behaviors associated with pollination events and should correlate with bee foraging effort. The unique acoustic structure of these buzzes [12] may allow non-lethal monitoring of bee behaviors associated with pollination success. Here, we test the potential for remote monitoring programs that capture acoustic signals of bee flight activity to inform pollination services.

For animal-pollinated plants, two main factors influence pollination success: pollinator abundance and pollination quality. Together, these attributes determine how much pollen is received and how effective that pollen is for ovule fertilization. Functional traits that influence bee pollination efficiency may have unique acoustic signatures, providing information on functional diversity and efficacy of bee assemblages. Tongue length alters the behavior and pollination efficiency of bees on long-tubed flowers [13–15], with short-tongued bees switching from pollination to robbing of nectar rewards [16]. Similarly, body size influences pollen transfer by changing the bee’s contact zone with the plant’s reproductive organs [17] and/or by altering flower visitation of other pollinators. For example, pollination efficiency of honey bees, *Apis mellifera*, increases in the presence of larger native bees, due to behavioral shifts in niche partitioning [18]. Because the first harmonic (hereafter, the characteristic frequency) of larger foragers have lower frequency [19], the size distribution of bee pollinators may correlate with buzz structure [12,20]. Pollination networks exhibit extensive redundancy and generalization [21–23], suggesting that the fates of plant hosts may depend more on guilds of pollinators than individual species. Indeed, functional diversity, rather than taxonomic diversity, enhances pollination services to several crop species [24–28]. Even for legumes that depend on bees of sufficient mass to “trigger” pollen release, pollination and fecundity depend more highly on activity of forager genera than individual species [29]. Thus, coarse-grained survey data lacking resolution at the species level may suffice for detecting the pollination deficits that accompany native bee declines.

Pollination ecologists supplement pollen delivery to flowers experimentally to test for pollen limitation and for the efficacy of natural pollinator service ([23,24] and references therein). Addition experiments are highly informative of the discrepancy between natural and optimal pollination levels, yet hand-pollination is time consuming, impractical for most woody species, and can reduce yield for plants with recessed stigmas or complex flowers due to inadvertent selfing or flower damage [30, 31]. Collecting pistils from senescing flowers for pollen enumeration has been used as an alternative (for example [32–34]) since cumulative pollen counts inform lifetime pollen receipt. This technique, too, is time consuming due to sample preparation and microscopy.

Here, we explore the potential for real-time, noninvasive acoustic monitoring of bee behaviors to simultaneously assess bee pollination services in multiple host populations. First, we conducted flight cage experiments and a literature review to determine the relationship between characteristic frequency and functional traits influencing pollination success (i.e., body size, tongue length). We then asked whether acoustic data aligned with visitation rates estimated from visual surveys, and we tested the efficacy of using buzz density (number of buzzes per hour) to estimate pollination services at the landscape scale for two bumble bee pollinated alpine forbs. Finally, we discuss the potential applications of this method for conservation, agricultural and basic research.

## Materials and methods

### Study system

Permission to conduct research at Ridge Long Term Ecological Research Site and Pennsylvania Mountain was granted by the Mountain Research Station, University of Colorado, and the Mountain Area Land Trust, respectively. We established methods for acoustic monitoring of bee pollination in alpine meadows of the Colorado Rocky Mountains during the 2014 and 2015 flowering seasons (June-August). Recordings of flight buzzes for bees of known species/caste were made in 2014 at the Niwot Ridge Long Term Ecological Research Site (Boulder County, Colorado, USA; 40°3.567'N, 105°37.000'W). Field tests of the efficacy of acoustic survey methods for remote tracking of bee abundance and pollination services were conducted in 2015 at Pennsylvania Mountain (Park County, Colorado, USA; 39°15.803'N, 106°8.564'W). In these Rocky Mountain field sites, forb reproductive success depends heavily on bumble bee pollination services [35]. We focused on the two main bumble bee species (*Bombus balteatus* and *B*. *sylvicola*) that are year-round residents and predominate above the timberline at our field sites [36–38].

### Characteristic frequency and pollinator functional traits

To determine if the characteristic frequency of a flight buzz varies with pollinator functional traits, we recorded flight buzzes of *Bombus balteatus* (*N* = 15) and *B*. *sylvicola* (*N* = 13) queens and workers. Individual bees were collected, cooled to torpor, identified to species and caste, and their wing length measured to estimate body size. Wing length is highly correlated with body size [39] and unlike mass is not influenced by pollen or nectar load. Each bee warmed-up for at least 15 minutes (or until torpor subsided) before foraging from a mixed patch of naturally co-occurring bumble bee host plants (e.g., *Trifolium dasyphyllum*, *Castilleja occidentalis*, *Polygonum bistorta*) in a 1x1x0.5m (WxLxH) flight cage. Using a Tascam US-1800 audio interface and Audacity v.2.0.5 (http://audacity.sourceforge.net/), five equidistant electret microphone capsules (OEM order number MPJA 30067; flat frequency response within ± 2 dB from 50 Hz to 10000 Hz) mounted at flower height (10–15 cm) recorded (32 bit/44.1 kHz sampling rate) flight buzzes for each bee as it foraged freely for up to six minutes. Bees were subsequently marked to prevent re-capture and released to their collection location.

We extracted buzz frequency measurements using the *Spectrum* function (Hamming Window, FFT size 8192) in Audacity v.2.0.5 (http://audacity.sourceforge.net/). Some recordings required removal of regular, sinusoidal electrical noise. A noise profile was identified for each track using a one second segment of pure noise and filtered from the track via the *Noise removal* function in Audacity. To better resolve the harmonic peaks despite some local variation in amplitude, we took a sliding average across 3 frequencies for each amplitude. We then determined the characteristic frequency for each buzz. Tongue length for each species and caste was measured as the combined length of the prementum and glossa from a published record [36].

We tested for a relationship between characteristic frequency and forager traits (wing length and tongue length) via mixed-model regression (*lme* in the *nlme* package [40]), with individual bee treated as a random effect. We report marginal r^2^ values (*r*.*squaredGLMM* in the *MuMIn* package [41]). The relationship between tongue length and characteristic frequency was also tested with wing length as a covariate via analysis of covariance (*lme* in the *nlme* package [40]) to determine if the relationship between tongue length and buzz frequency was an artifact of a correlated trait, body size. Reported results reflect type III sum of squares (*anova*.*lme* in the *nlme* package [40]).

We compiled characteristic frequency and trait measurements from published records for queens and workers of an additional 17 bumble bee species (9 species with two casts reported). We searched Google Scholar in June 2016 for characteristic frequencies of flight buzzes using the following search parameters: “bombus flight buzz frequency” and “bombus wing beat frequency”. We included only studies that recorded buzzes in free flight and in natural or semi-natural conditions. We then searched for tongue length measurements for each species using the following: “[species name] tongue length” and “[species name] proboscis length”. Tongue length was measured as the combined length of the glossa and the prementum; all other measures were excluded. If more than one value was found for the tongue length or the frequency of the flight buzz (S1 Table), we calculated the weighted mean based on the sample size for each value [42]. We tested for a relationship between buzz frequency and tongue length across all species and casts (*lm* in the R Statistical Software *stats* package [43]). The buzz frequency distribution was left skewed due to relatively few species with high values, so the data were log transformed to meet assumptions of normality [35].

### Visual vs. acoustic estimates of pollinator abundance

Acoustic surveys and simultaneous visual observations took place during peak flowering (24–31 July 2015) for two bumble bee pollinated plant species, the alpine clovers *Trifolium dasyphyllum* and *T*. *parryi* [29]. Surveys were conducted in three, circular 0.01 km^2^ area plots spaced 255–784 m apart along an altitudinal gradient (3659m, 3698m, and 3735m [35]). *Trifolium dasyphyllum* and *T*. *parryi* were dominant floral resources within these plots, accounting for 39–67% and 21–45% of daily flowering, respectively. Other bumble bee hosts included *Phacelia sericea* (3–6%), and *Mertensia lanceolata* (1–8%). These plants do not require sonication (vibration of the anthers to induce pollen release), and no buzz pollinated plants were blooming during these observations. Therefore, all buzzes were inferred to be flight buzzes. Manual annotations of the buzzes yielded only one potential pollination buzz out of 11,364 buzzes recorded. Five electret microphone capsules mounted at plant height were placed evenly apart in areas of dense flowering for *T*. *dasyphyllum* and *T*. *parryi*. Acoustic signals were recorded with the Awesome Voice Recorder (AVR version 3.5; Newkline Co., Ltd) application at a sampling rate of 11,025 kHz/48 kbps (compressed MP3 format) installed on iPad Mini’s **©** (Apple Inc., Model MF433LL/A; IOS version 7.1). Recordings were made under clear skies between 0800–1400 (MST) for a total of 18.5 observer h (5.5–7 h per site). Visual surveys were conducted over 15 min intervals during alternating hours concurrent with the acoustic surveys. Observers walked an even serpentine pattern across the flowering area, counting all bumble bees within approximately 50 cm. Each bee observed foraging within that 50 cm radius was counted as one individual, regardless of the number of plants she visited.

To detect buzzes within field recordings, we developed a Computational Auditory Scene Analysis (hereafter CASA) approach to processing the signals [44]. CASA implements the principles of auditory scene analysis (ASA) via computer algorithms that can “listen” in a similar manner as humans [45]. We extended an approach for using spectral clustering [45] by adding *focal templates* as a means of specifying our sound source of interest (i.e., buzzing). The focal templates pass along (bring to the foreground of attention) potential time-frequency elements that may comprise a buzz (i.e., those in harmonic relation) to a spectral clustering stage. Time-frequency elements not conforming to the expected pattern, likely not belonging to a buzz, are pushed to the background and ignored [44]. After the focal template is applied, spectral clustering is performed to partition the focused spectrogram into three clusters (allowing for more than one buzz to be identified as separate clusters). First, an affinity matrix is constructed by determining the similarity between the remaining time-frequency bins. K-means clustering is applied to the smallest three eigenvectors of the affinity matrix to partition time-frequency bins into clusters. If the density of time-frequency points in the spectrogram of a cluster is greater than or equal to 1.0, the segment of time is reported as a prospective buzz. A cluster not containing a buzz will contain time-frequency bins corresponding to noise. To mitigate the effect of low signal to noise ratio, the entire cluster of the prospective buzz is then convolved with a 20-element vector and summed across frequency into a one-dimensional “smashed cluster” vector in the time domain. If the peak within the resulting “smashed cluster” reaches 1.0, the cluster is recorded as a buzz.

The automated buzz counts were compared to manual (auditory and visual) inspections of the spectrograms in twelve randomly selected segments of recordings across the three sites. We tested for a positive relationship (1-tailed *P*) via correlation (*cor* in the *stats* package [43]). We then tested the reliability of acoustic estimates relative to traditional visual surveys of bee abundance via Pearson’s correlation [46].

### Quantifying pollination services via acoustic surveys

Pollinator exclusion experiments were conducted concurrently with bumble bee surveys to assess pollination services for *Trifolium dasyphyllum* and *T*. *parryi* [29]. Plants of both species are obligately outcrossing and depend on visits from queens of *B*. *sylvicola* and *B*. *balteatus* for seed production. In each plot, twenty-five, randomly selected inflorescences were left open to bumble bee pollinators, and 25 were caged with hardware cloth cylinders of 0.6 cm mesh size to exclude queen bumble bees. Following fruit set, infructescences were collected, and seeds per infructescence counted. We tested whether the difference in seed set with and without queen bumble bees was greater than zero via one-tailed *t*-test [46] to determine if bumble bees contributed to seed set. To test whether acoustic buzz counts explain variation in seed set, we conducted an analysis of covariance assessing the relationship between buzz density and seed set, with plant species as a covariate [46]. All analyses were conducted using plot means for seed production.

## Results

### Characteristic frequency and pollinator functional traits

**Fig 2:**
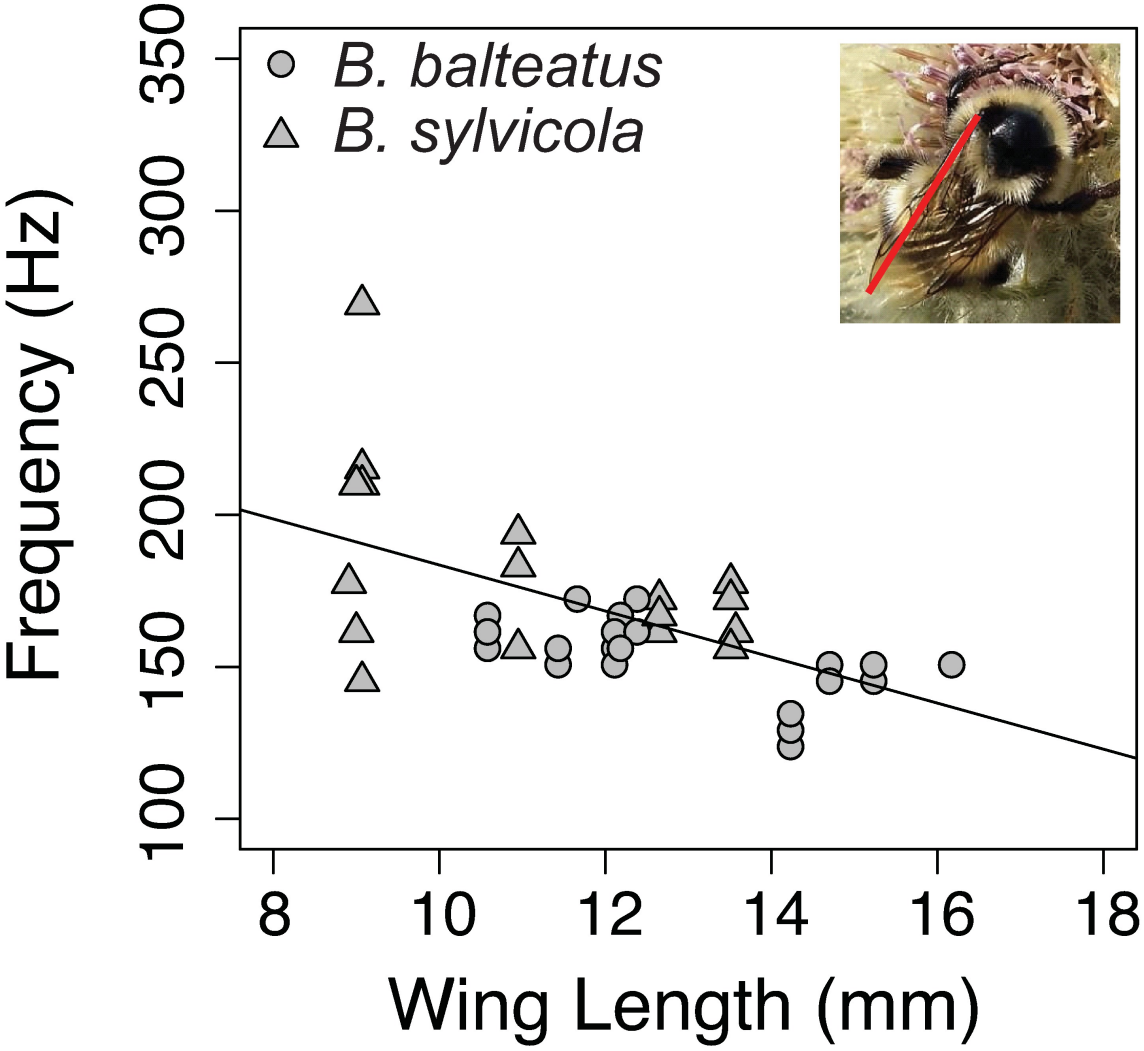
Characteristic frequency of flight buzzes for workers and queens of two alpine bumble bee species. Buzz frequency was negatively related to wing length (indicated by the red line) (y = 259.2–7.57x), an estimate of body size that is not biased by pollen and nectar loads of the bees.

**Fig 3:**
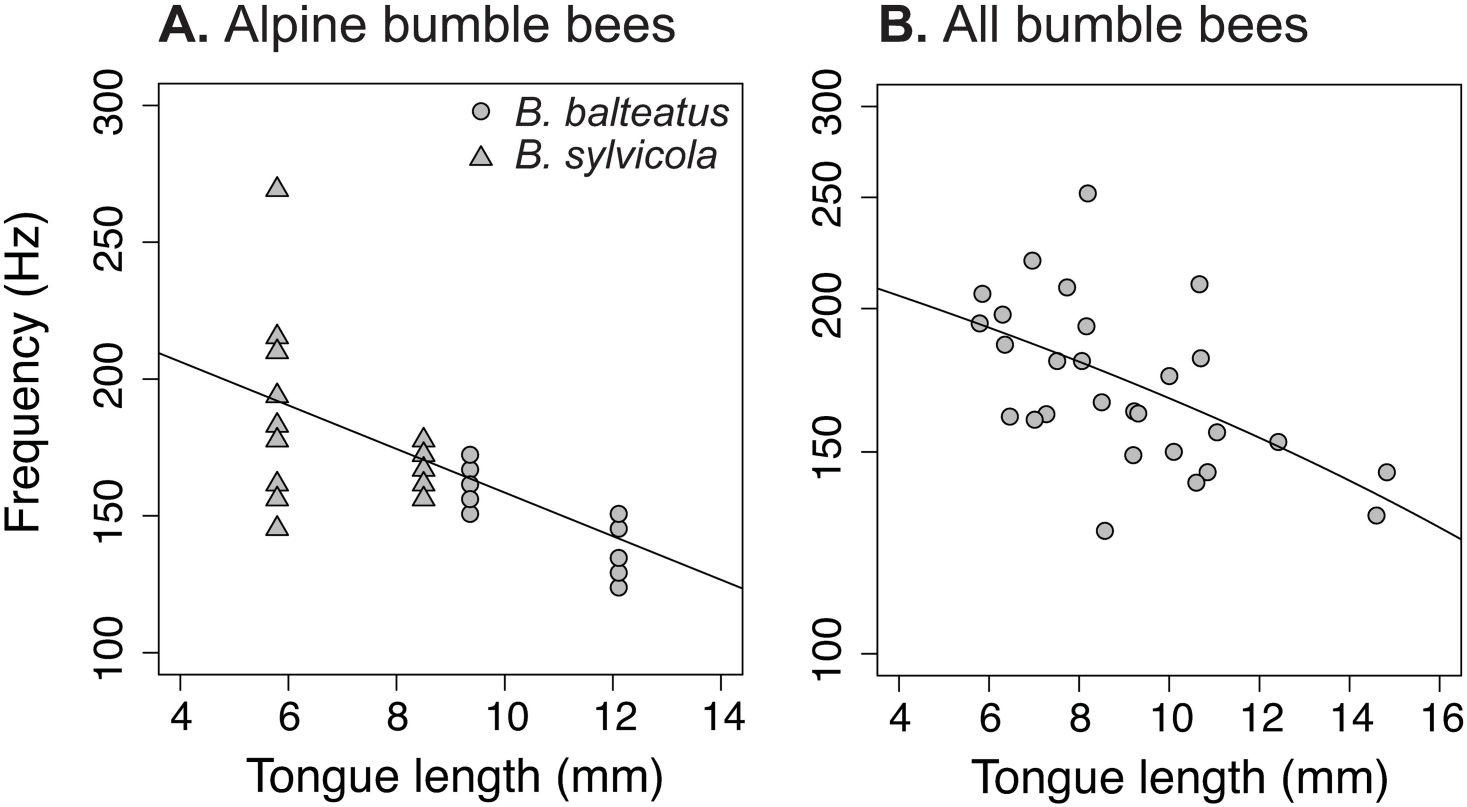
Characteristic frequency is related to bumble bee tongue length. (A) Variation in characteristic frequency for two alpine bumble bees, *Bombus balteatus* and *B*. *sylvicola* is explained by tongue length (y = 238.2–7.96x), indicating that acoustic signals reflect functional trait diversity. (B) Literature reports of tongue length for workers and queens of 17 bumble bee species also correlate with characteristic frequency (y = 5.48 + e^-0.376x^). Tongue length measurements correspond to the weighted means of published accounts for each species and caste combination (S1 Table).

Characteristic frequency of workers and queens of *Bombus balteatus* and *B*. *sylvicola* in flight was correlated with wing length (r^2^_m_ = 0.439, *F*_1,16_ = 24.40, *P* < 0.0001; Fig 2), an estimate of body size, and tongue length (r^2^ = 0.523, *F*_1,16_ = 39.47, *P* < 0.0001; Fig 3A). Similarly, tongue length predicted characteristic frequency in a broader sample of workers and queens of 17 bumble bee taxa reported in the literature (r^2^ = 0.302, *F*_1,26_ = 11.22, *P* = 0.0025; Fig 3B). This relationship holds (r^2^ = 0.525, *F*_1,15_ = 6.62, *P* = 0.021) for alpine bumble bees when wing length is included as a covariate, indicating that body size does not fully explain the relationship between tongue length and buzz frequency.

### Visual vs. acoustic estimates of pollinator abundance

**Fig 4:**
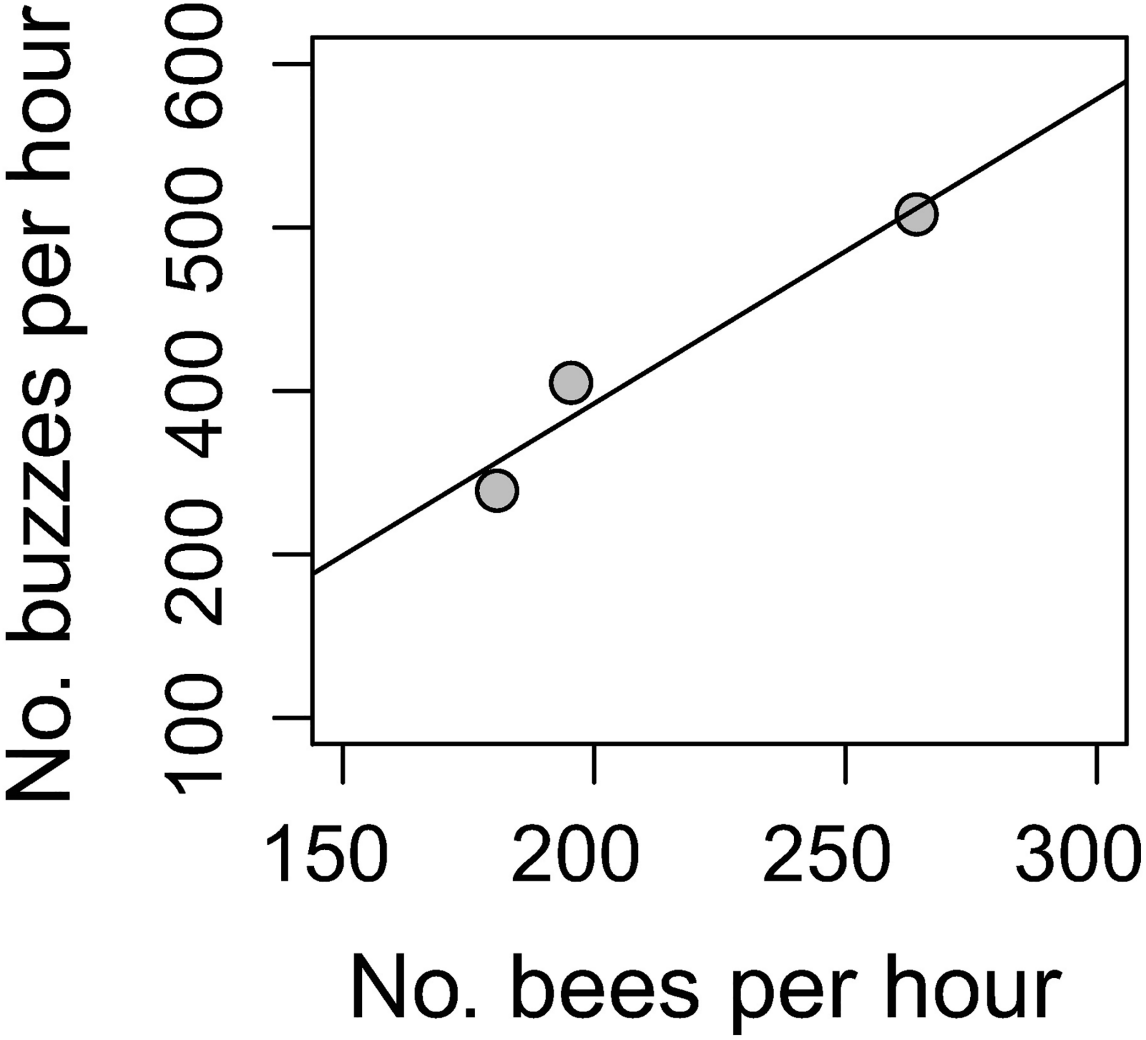
The number of buzzes recorded is correlated with the number of bees counted during visual observations.

Automated buzz counts were correlated with manual buzz counts (*r*_1,2_ = 0.956, *P* = 0.095). A high correlation coefficient supports the use of the automated procedure for quantifying buzz abundance, despite a marginally significant *P-value* which is the product of the low sample size (N = 3 plots). Additionally, acoustic estimates of buzz abundance correlated with traditional survey counts (*r*_1,2_ = 0.973, *P* = 0.0011; Fig 4), indicating that less time and labor-intensive remote monitoring methods using acoustic signals provide robust results. Buzz counts were higher (417.3 ± 16.3 buzzes per hour, mean ± SE) than traditional counts (213.5 ± 31.1 bees per hour, mean ± SE), presumably because buzz counts, but not visual observations, accumulated with flights of the same forager between sequentially visited host plants and because individual observers will miss bees foraging simultaneously near spatially isolated microphones within a plot.

### Quantifying pollination services via acoustic surveys

**Fig 5:**
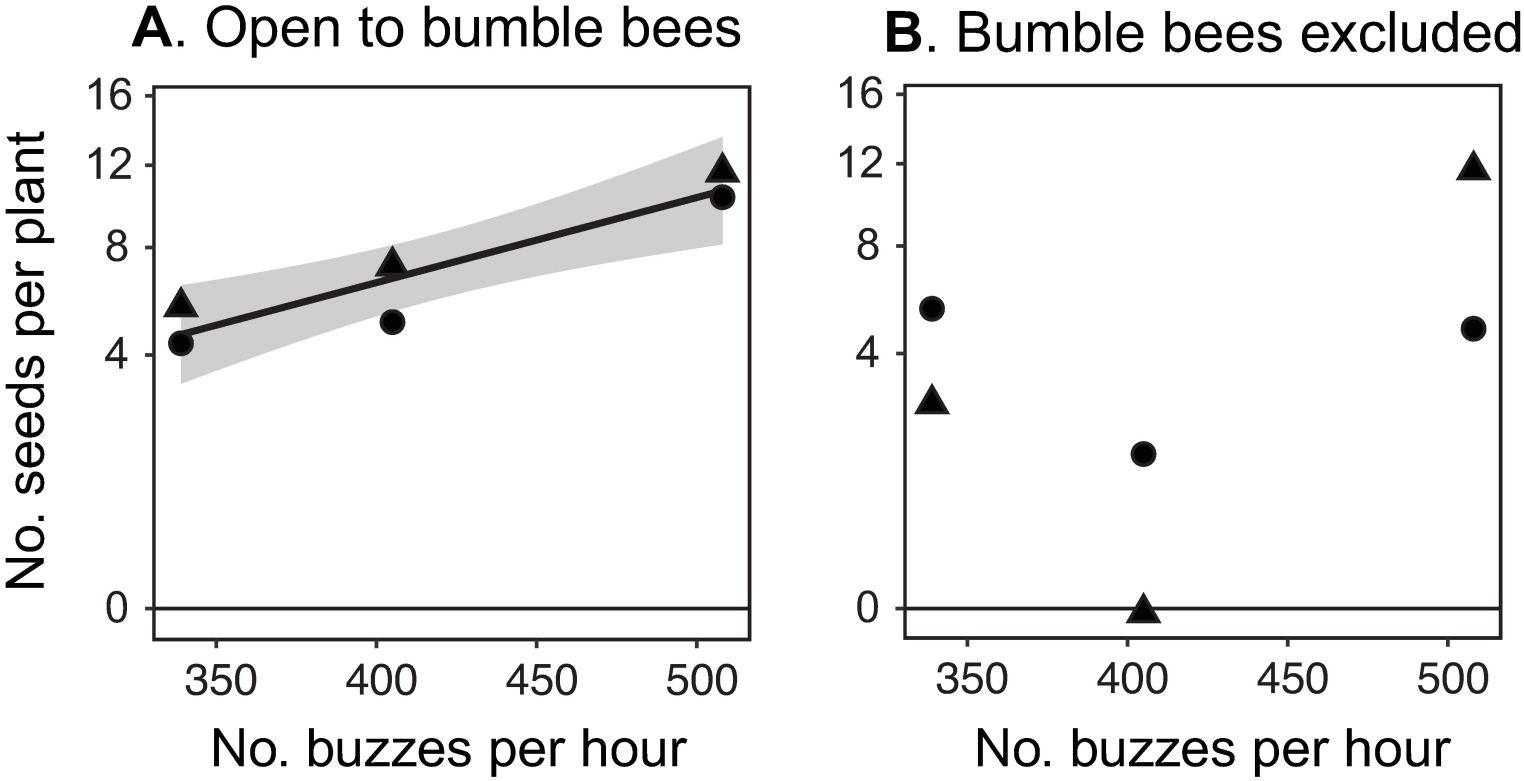
Acoustic surveys predict pollination services in two alpine flowering forbs. When flowers of *Trifolium dasyphyllum* (circles) and *T. parryi* (triangles) are left open to bumble bee visitors, buzz abundance (A) predicts the average number of seeds per plant in each of three 0.01 km^2^ plots. When bumble bee pollinators are excluded, buzz abundance fails to predict seed set (B), illustrating that acoustic surveys document pollination services by bumble bees. Gray shading around the line represents the standard error of the mean [47].

Acoustic surveys of bee abundance provide an accurate estimate of pollination services to populations of two bumble bee pollinated clovers. Bumble bee exclusion indicated that bees contributed to seed of both *T*. *dasyphyllum* and *T*. *parryi*. When bumble bees were allowed to visit flowers, an average of 3.89 more seeds were set per plant than when they were excluded (*t*_5_ = 2.33, 1-tailed *P* < 0.0334). Acoustic estimates of bee abundance predicted seed set (average number of seeds per plant in each site) when bumble bees were allowed to visit flowers (*F*_1,3_ = 63.56, *P* = 0.0041; Fig 5A), but not when bumble bees were excluded (*F*_1,3_ = 0.64, *P* = 0.48; Fig 5B). Accrual curves of seed production with buzz density did not differ between species (open to bumble bee visitors: *F*_1,3_ = 7.02, *P* = 0.077; bumble bee excluded: *F*_1,3_ = 0.05, *P* = 0.84).

## Discussion

In light of the agricultural and ecological importance of bee pollinators, scientists and policy makers are calling for widespread and intensive population monitoring [48–50]. Tracking dynamic populations of mobile organisms, however, requires formidable monetary and personnel resources [7,48,49]. By continuously monitoring the soundscapes of a region, automated, acoustic surveys could provide farmers and scientists with biologically relevant data at a rapid pace (S1 Fig). Acoustic surveys offer the opportunity for real-time monitoring over broad scales matching the foraging ranges, population dynamics and shifting distributions of bee pollinators. The technology for acoustic data collection and processing is nimble, low cost, and suited to remote locations. Here, we show that relatively coarse-grained acoustic signals associated with bee flight buzzes can be leveraged to monitor pollinator activity and pollination services at the landscape scale for two bumble bee pollinated wildflowers.

Pollination services are improved when diversity is high [26,28,51–53] and partner functional traits exhibit morphological matching [54–56]. Our results indicate that acoustic signals capture diversity in traits that mediate pollination success. Specifically, bee body size and tongue length predict characteristic frequency (Figs 2 and 3), reflecting the phenotypic landscape of the community. This functional diversity relates directly to pollination services by modifying the phenological complementarity between mutualists, niche partitioning among pollinators, and within-plant pollinator foraging strategies [53]. Indeed, functional trait diversity better predicts crop yield than taxonomic diversity [24,25]. While taxonomic specificity is necessary when monitoring a particular species of conservation concern, these results suggests that coarse-grained data may suffice for tracking pollination services, particularly in agricultural settings.

The concordance between visual and acoustic estimates of bee activity (Fig 4) indicates that acoustic signals can be harnessed to survey buzzing insects in natural environments. Eavesdropping or monitoring the acoustic signals exchanged by individuals has revolutionized detection of mobile and cryptic organisms such as birds, frogs, etc. [10,57]. Acoustic surveys free up capital (i.e., expertise, financial, biological) to monitor multiple, inaccessible and inhospitable habitats simultaneously [58,59] while minimizing human exposure to potentially hazardous conditions. Traditional surveys, even when robustly implemented, provide snapshots in time, which are susceptible to fluctuating weather conditions and population sizes. Acoustic surveys circumvent this weakness and, in some systems, provide more reliable data, more rapidly than human-based techniques [59,60]. While scientists have surveyed vibrations in plant tissues to monitor insects pests, airborne signals are rarely used to monitor insect populations [18,61]. Our algorithm accurately and rapidly detected flight buzzes, allowing near instantaneous indicators of pollinator activity (S1 Fig) and suggesting that surveys of airborne signals may similarly transform bee monitoring programs. Real-time assessment of buzz diversity and density would allow farmers to make informed decisions on whether to supplement wild pollinators with managed species and to assess benefits of interventions aimed at enhancing pollination services (e.g., cover crops, hedge rows, no-till practices).

Because they can be differentiated from pollination buzzes [12], flight buzzes capture potential outcrossing events that reflect pollination services. Flight buzz density was predictive of wildflower reproductive success (Fig 5) and could be used to test hypotheses on the causal basis of pollen limitation. For instance, continuous surveys provide the necessary data to distinguish between two competing theories: that plants are adapted to average pollination conditions [62] or stochasticity in their pollination environment [31,63]. Similarly, acoustic data could illuminate whether selection favors individuals with the capacity to sense and respond to buzz density. Plants sense vibrations from herbivores, responding to threats by initiating defense mechanisms [64]. If plants have similar response mechanisms to buzzes, they may respond to the dearth of or identity of available pollinators by phenotypic plasticity in attraction traits, such as nectar production or scent chemistry. The strong fitness consequences of buzz density (Fig 5) suggest that such a strategy could be selectively advantageous.

## Conclusion

Our results indicate that eavesdropping on bee flight buzzes provides landscape scale estimates of pollinator activity, functional diversity, and pollination services while minimizing observer disturbance. By circumventing weaknesses of traditional survey techniques, acoustic surveys monitor bees at biologically relevant scales while simultaneously creating opportunities for broader stakeholder involvement (S1 Fig). Inexpensive technology and automated processing of acoustic recordings produces tractable records [65], making the technique ideal for citizen involvement. Given seasonal and inter-annual variation in bee populations, frequent, noninvasive surveys are necessary to reliably track population fluctuations and pollination services. Passive monitoring of the buzz soundscapes provides the opportunity to monitor bees in an efficient and cost-effective way, potentially improving our ability to understand the complex nature and global implications of bee declines.

## Supporting information

S1 Fig Acoustic monitoring may improve farmer and biologist response times to pollinator population deficits. Acoustic signals reflect pollinator abundance and functional traits that predict pollination services. Leveraging these signals, pollinator deficits can be identified quickly, allowing farmers to supplement wild pollinators with domesticated bee colonies. Inexpensive, long-term monitoring provides population estimates in many locations simultaneously, allowing conservation biologists to disentangle the effects of multiple environmental stressors. Researchers can explore fundamental questions concerning communication among organisms and ‘eavesdropping’ by non-target organisms (such as arachnid predators or pollinator host plants that may respond to flight buzzes as indicators of prey availability or pollination quality).
(TIF)

S1 Table References for characteristic frequency of flight buzzes and tongue length measurements collated from the literature. The mean weighted by sample size was calculated for any species and caste with more than value for characteristic frequency or tongue length.
(DOCX)
