# Supplementary figures and images for: Flight of the bumble bee: Buzzes predict pollination services
Source: PLoS One. 2017 Jun 7;12(6):e0179273. doi: 10.1371/journal.pone.0179273 (PMC5462477; doi:10.1371/journal.pone.0179273)

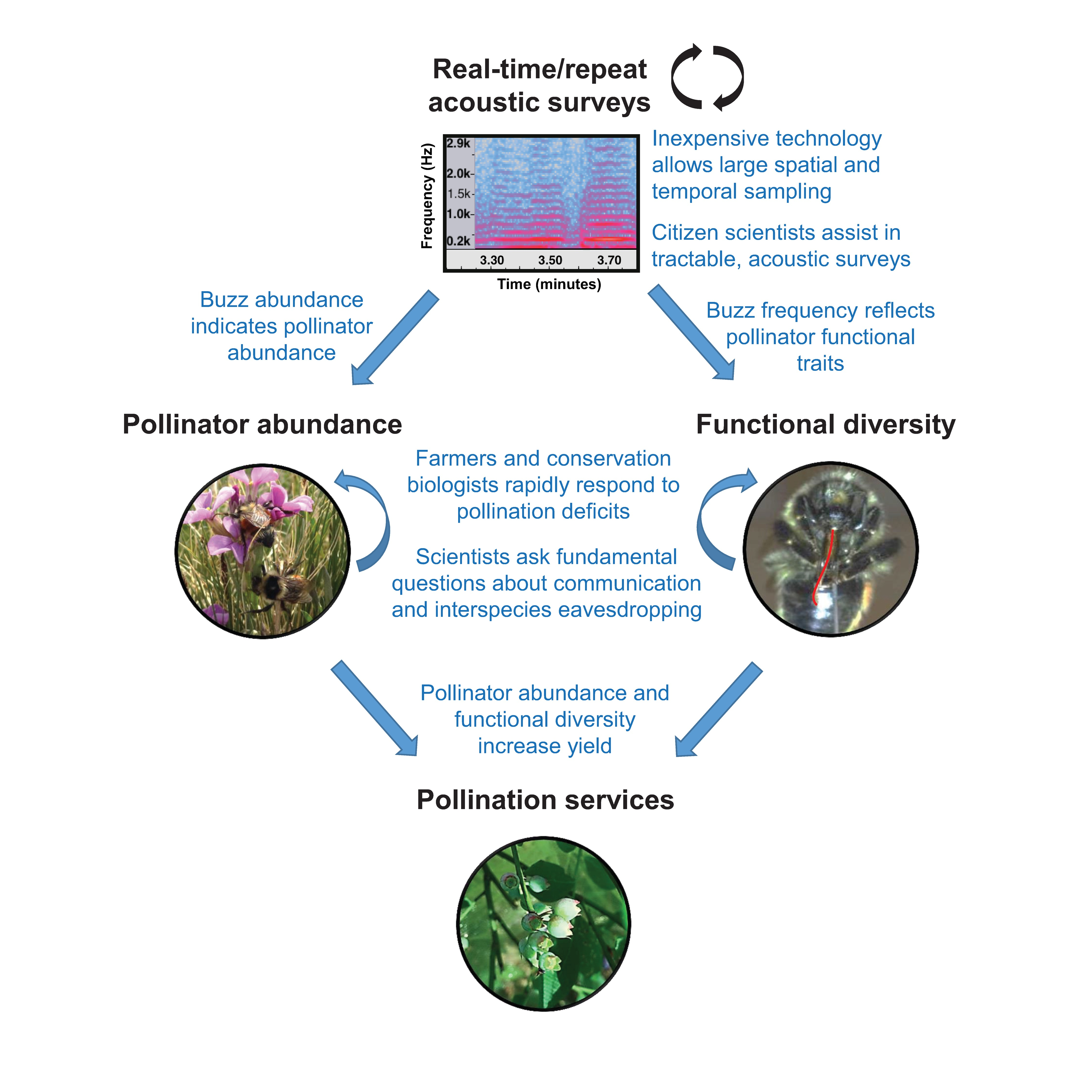

Supplement: S1 Fig — Acoustic signals reflect pollinator abundance and functional traits that predict pollination services. Leveraging these signals, pollinator deficits can be identified quickly, allowing farmers to supplement wild pollinators with domesticated bee colonies. Inexpensive, long-term monitoring provides population estimates in many locations simultaneously, allowing conservation biologists to disentangle the effects of multiple environmental stressors. Researchers can explore fundamental questions concerning communication among organisms and ‘eavesdropping’ by non-target organisms (such as arachnid predators or pollinator host plants that may respond to flight buzzes as indicators of prey availability or pollination quality). (TIF) [file pone.0179273.s001.tif]
