# Supplementary material for: Flight of the bumble bee: Buzzes predict pollination services
Source: PLoS One. 2017 Jun 7;12(6):e0179273. doi: 10.1371/journal.pone.0179273 (PMC5462477; doi:10.1371/journal.pone.0179273)
Supplement: S1 Table — The mean weighted by sample size was calculated for any species and caste with more than value for characteristic frequency or tongue length. (DOCX) [file pone.0179273.s002.docx]

**Supporting Information**

**S2 Table.**  **References for characteristic frequency of flight buzzes and tongue length measurements collated from the literature.** The mean weighted by sample size was calculated for any species and cast with more than value for characteristic frequency or tongue length.

| **Species** | **Cast** | **Characteristic frequency: Author(s)** | **Tongue length: Author(s)** |
| --- | --- | --- | --- |
| *B. balteatus* | queen | Miller-Struttmann et al. | Medler (1962), Macior (1974), Ranta (1982) |
|  | worker | Miller-Struttmann et al. | Medler (1962), Macior (1974) |
| *B. festivus* | worker | Corbet and Huang (2014) | Wang & Li (2005) |
| *B. friseanus* | worker | Corbet and Huang (2014) | Williams et al. (2009) |
| *B. hortorum* | queen | Gradišek et al. (2016) | Ranta (1982) |
|  | worker | Gradišek et al. (2016) | Pekkarinen (1979), Goulson et al. (2005) |
| *B. humilis* | queen | Gradišek et al. (2016) | Medler (1962), Ranta (1982) |
|  | worker | Gradišek et al. (2016) | Medler (1962), Goulson et al. (2005) |
| *B. hypnorum* | queen | Gradišek et al. (2016) | Pekkarinen (1979), Ranta (1984) |
|  | worker | Gradišek et al. (2016) | Ranta (1984), Goulson et al. (2008) |
| *B. impatiens* | worker | Switzer et al. (2016) | Medler (1962) |
| *B. jonellus* | worker | Gradišek et al. (2016) | Ranta (1984), Goulson et al. (2005), Goulson et al. (2008) |
| *B. lapidarius* | queen | Gradišek et al. (2016) | Pekkarinen (1979), Ranta (1984) |
|  | worker | Gradišek et al. (2016) | Pekkarinen (1979), Ranta (1984), Goulson et al. (2005), Goulson et al. (2008) |
| *B. lucorum* | queen | Gradišek et al. (2016) | Medler (1962), Ranta (1982), Ranta (1984) |
|  | worker | Gradišek et al. (2016) | Medler (1962), Pekkarinen (1979), Ranta (1984), Goulson et al. (2005), Goulson et al. (2008) |
| *B. pascuorum* | queen | Gradišek et al. (2016) | Pekkarinen (1979) |
|  | worker | Gradišek et al. (2016) | Pekkarinen (1979), Goulson et al. (2005), Goulson et al. (2008) |
| *B. pratorum* | queen | Gradišek et al. (2016) | Medler (1962), Ranta (1982), Ranta (1984) |
|  | worker | Unwin (1984), Gradišek et al. (2016) | Medler (1962), Pekkarinen (1979), Ranta (1984), Goulson et al. (2005), Goulson et al. (2008) |
| *B. ruderarius* | worker | Gradišek et al. (2016) | Ranta (1984), Goulson et al. (2005) |
|  | queen | Gradišek et al. (2016) | Pekkarinen (1979), Ranta (1984) |
| *B. sylvarum* | queen | Gradišek et al. (2016) | Medler (1962), Ranta (1982), Ranta (1984) |
|  | worker | Gradišek et al. (2016) | Medler (1962), Ranta (1984), Goulson et al. (2005) |
| *B. sylvicola* | queen | Miller-Struttmann et al. | Macior (1974) |
|  | worker | Miller-Struttmann et al. | Macior (1974) |
| *B. terrestris* | queen | Gradišek et al. (2016) | Pekkarinen (1979) |
| *B. terricola* | worker | Morgan et al. (2016) | Medler (1962) |

**Literature Cited**

Corbet SA, Huang SQ. Buzz pollination in eight bumblebee-pollinated *Pedicularis* species: does it involve vibration-induced triboelectric charging of pollen grains? Ann Bot. 2014;114: 1665–1674. doi:10.1093/aob/mcu195

Goulson D, Hanley ME, Darvill B, Ellis JS, Knight ME. Causes of rarity in bumblebees. Biol Conserv. 2005;122: 1–8. doi:10.1016/j.biocon.2004.06.017

Goulson D, Lye GC, Darvill B. Diet breadth, coexistence and rarity in bumblebees. Biodivers Conserv. 2008;17: 3269–3288. doi:10.1007/s10531-008-9428-y

Gradišek A, Slapničar G, Šorn J, Luštrek M, Gams M, Grad J. Predicting species identity of bumblebees through analysis of flight buzzing sounds. Bioacoustics. 2016;4622: 1–14. doi:10.1080/09524622.2016.1190946

Macior LW. Pollination ecology of the front range of the Colorado Rocky Mountains. Melanderia. 1974;15: 1–59.

Medler J. Morphometric studies on bumble bees. Ann Entomol Soc Am. 1962;55: 212–218.

Morgan T, Whitehorn P, Lye GC, Vallejo-Marín M. Floral sonication is an innate behaviour in bumblebees that can be fine-tuned with experience in manipulating flowers. J Insect Behav. 2016;29: 233–241. doi:10.1007/s10905-016-9553-5

Pekkarinen A. Morphometric, colour and enzyme variation in bumblebees (Hymenoptera, Apidae, *Bombus*) in Fennoscandia and Denmark. Acta Zool Fenn. 1979;158: 1–60.

Ranta E, Lappalainen K, Miettinen H. Foraging dynamics of two bumblebee species (*lucorum* and *lapidarius*) during one summer. Ann Zool Fenn. 1984;21: 77–88.

Ranta E. Species structure of North European bumblebee communities. Oikos. 1982;38: 202–209. doi:10.2307/3544020

Switzer CM, Hogendoorn K, Ravi S, Combes SA. Shakers and head bangers: differences in sonication behavior between Australian *Amegilla murrayensis* (blue-banded bees) and North American *Bombus impatiens* (bumblebees). Arthropod Plant Interact. Springer Netherlands; 2016;10: 1–8. doi:10.1007/s11829-015-9407-7

Unwin DM, Corbet SA. Wingbeat frequency, temperature, and body size in bees and flies. Physiol Entomol. 1984;9: 115–121. doi:10.1111/j.1365-3032.1984.tb00687.x

Wang H, Li D-Z. Pollination Biology of Four Pedicularis Species (Scrophulariaceae) in Northwestern Yunnan, China. Source Ann Missouri Bot Gard. 2005;92: 127–138.

Williams P, Colla S, Xie Z. Bumblebee vulnerability: common correlates of winners and losers across three continents. Conserv Biol. 2009;23: 931–940. doi:10.1111/j.1523-1739.2009.01176.x
